# Audiovisual integration in near and far space: effects of changes in distance and stimulus effectiveness

**DOI:** 10.1007/s00221-015-4248-2

**Published:** 2015-03-19

**Authors:** N. Van der Stoep, S. Van der Stigchel, T. C. W. Nijboer, M. J. Van der Smagt

**Affiliations:** 1 Department of Experimental Psychology, Helmholtz Institute, Utrecht University, Heidelberglaan 1, 3584 CS Utrecht, The Netherlands; 2 Brain Center Rudolf Magnus, and Center of Excellence for Rehabilitation Medicine, De Hoogstraat Rehabilitation, University Medical Center Utrecht and De Hoogstraat Rehabilitation Center, Utrecht, The Netherlands

**Keywords:** Multisensory, Crossmodal, Integration, Audiovisual, Depth, Space

## Abstract

A factor that is often not considered in multisensory research is the distance from which information is presented. Interestingly, various studies have shown that the distance at which information is presented can modulate the strength of multisensory interactions. In addition, our everyday multisensory experience in near and far space is rather asymmetrical in terms of retinal image size and stimulus intensity. This asymmetry is the result of the relation between the stimulus-observer distance and its retinal image size and intensity: an object that is further away is generally smaller on the retina as compared to the same object when it is presented nearer. Similarly, auditory intensity decreases as the distance from the observer increases. We investigated how each of these factors alone, and their combination, affected audiovisual integration. Unimodal and bimodal stimuli were presented in near and far space, with and without controlling for distance-dependent changes in retinal image size and intensity. Audiovisual integration was enhanced for stimuli that were presented in far space as compared to near space, but only when the stimuli were not corrected for visual angle and intensity. The same decrease in intensity and retinal size in near space did not enhance audiovisual integration, indicating that these results cannot be explained by changes in stimulus efficacy or an increase in distance alone, but rather by an interaction between these factors. The results are discussed in the context of multisensory experience and spatial uncertainty, and underline the importance of studying multisensory integration in the depth space.

**Electronic supplementary material:**

The online version of this article (doi:10.1007/s00221-015-4248-2) contains supplementary material, which is available to authorized users.

## Introduction

Integration of information from different senses has been extensively studied in the last few decades (see Stein et al. 2010, Figure 6; Murray et al. 2013; Van der Stoep et al. 2014, Figure 1). Several rules, or principles, have emerged from neurophysiological studies that are known to be important for multisensory integration (MSI) to occur (or to enhance it, e.g., King and Palmer 1985; Stein and Meredith 1993). Typically, the effects of multisensory stimulation are more pronounced (e.g., shorter response times, RT) when stimuli from different senses are *spatially* and *temporally aligned* (Holmes and Spence 2004; though see Spence 2013; see Stein and Stanford 2008, for a review). In addition, the principle of *inverse effectiveness* states that the benefit of multisensory interactions is largest when responses to unisensory stimuli are weak (e.g., Meredith and Stein 1983; Holmes 2007). In neurophysiological studies, relative increases in multisensory responses with decreasing stimulus intensity have been observed in multisensory neurons (in terms of relative increase in spike rates in single- or multi-unit recordings; e.g., Alvarado et al. 2007).

In humans, however, results from behavioral studies have shown conflicting findings, mainly with respect to the spatial rule (Spence 2013) and the principle of inverse effectiveness (Holmes 2007, 2009). Concerning the principle of inverse effectiveness, several behavioral studies have reported inconsistent results regarding the relation between stimulus intensity (or signal-to-noise ratio) and either the amount of multisensory response enhancement (i.e., faster or more accurate responses to multisensory stimuli in comparison with responses to unimodal stimuli) or MSI (i.e., enhancement beyond what would be expected based on an independent channel model, Miller 1982, 1986; e.g., Leone and McCourt 2013; Stevenson et al. 2012; Ross et al. 2007). For example, Leone and McCourt (2013) observed larger benefits of MSI (i.e., shorter RTs) when multisensory stimuli were composed of unimodal stimuli of *higher* intensity as compared to when stimuli were of lower intensity. These studies demonstrate that it is difficult to consistently replicate some of the neurophysiological observations regarding the principle of inverse effectiveness in behavioral studies in humans.

Although the majority of behavioral studies in humans have looked into the principles governing MSI at a fixed distance from the observer, there are several reasons to believe that the integration of information from certain sensory modalities is more or less effective depending on the region of space from which multisensory stimuli are presented (for review, see Van der Stoep et al. 2014). For example, multisensory interactions involving touch are more pronounced in near (or peripersonal space, the space directly surrounding different body parts; e.g., Rizzolatti et al. 1981; Occelli et al. 2011) as compared to far space (the space further away from the body that is out of reach). In contrast, audition and vision seem to be the dominant senses in far space (or action-extrapersonal space; Previc 1998). This difference in distance-based sensory modality dominance may not be surprising when thinking of the differing behavioral functions that are related to near and far space. Grasping and manipulating objects require direct contact between the body and the environment and are typical behavioral functions bound to near space. One of the dominant functions in far space is spatial orienting, a much more audiovisual-based function which does not necessitate contact between the environment and the body in the same way as, for example, grasping. Furthermore, different neuroanatomical systems seem to be involved in the processing of near (e.g., Graziano and Gross 1994; Graziano et al. 1999) and far space (e.g., Aimola et al. 2012; Committeri et al. 2007; for review see Previc 1998). Brain regions coding near space seem to be more related to audiotactile/visuotactile and motor processing (e.g., Serino et al. 2011), whereas brain regions coding far space seem to be more related to spatial orienting and audiovisual integration (Previc 1998).

In addition to possible distance-based sensory modality dominance, a change in the distance from which stimuli are presented also changes the arrival time of auditory and visual stimuli. For instance, increasing the distance between audiovisual stimuli and the observer increases the difference in arrival times of auditory and visual stimuli that are caused by the difference in the speed of light and sound. It has been shown that the difference in the speed of light and sound is taken into account when judging the simultaneity of audiovisual sources in depth (e.g., Alais and Carlile 2005; Arnold et al. 2005; Sugita and Suzuki 2003). This effect, however, seems to depend on whether estimating external temporal alignment is task relevant (see also Heron et al. 2007). These studies indicate that fairly accurate estimates about the distance of multisensory stimuli can be made, possibly as a result of prior multisensory experience with the environment.

Finally, increasing the distance between a multisensory stimulus and an observer also decreases the retinal image size and intensity of visual stimuli (e.g., through the inverse-square rule, Warren 1963) and the intensity and direct-to-reverberant ratio of auditory stimuli (Alais and Carlile 2005; Bronkhorst and Houtgast 1999). Because of this relation between distance and stimulus properties and audiovisual dominance in far space, audiovisual integration may be specifically enhanced when an increase in distance and a decrease in retinal image size and stimulus intensity go hand in hand.

In order to investigate the possible interplay between distance (region of space), retinal image size, and stimulus intensity, audiovisual stimuli of two different stimulus intensities and sizes were presented both in near and far space. A near space condition was used with an audiovisual stimulus of a certain size and intensity (*Near High*) and a far space condition in which the same stimulus was presented at a larger distance from the observer (*Far Low*). To be able to disentangle the influence of distance, retinal image size and intensity, and their interaction on MSI, a condition was added in which the audiovisual stimulus in near space had the same decrease in retinal image size and intensity (*Near Low*) as the stimulus in the *Far Low* condition. To balance the design, a final condition was constructed (*Far High*) which consisted of the same near space stimulus (*Near High*) at a larger distance, but corrected for retinal image size and intensity (*Far High*).

Thus, by comparing these four conditions, the effects of changes in distance, in stimulus efficacy, and distance-related changes in stimulus efficacy on audiovisual integration could be investigated. We hypothesized that audiovisual integration may be more pronounced in far space, given the potential dominance of audiovisual information in far space, and the lawful relation between changes in distance and changes in stimulus efficacy.

## Methods

### Participants

Twenty participants were tested in the experiment (11 male, mean age = 24.58 years, SD = 3.55). All participants reported to have normal or corrected-to-normal visual acuity and normal hearing. The experiment was conducted in accordance with the Declaration of Helsinki. All participants signed an informed consent form before taking part in the study and received either monetary compensation or course credits for their participation.

### Apparatus and stimuli

**Fig. 1:**
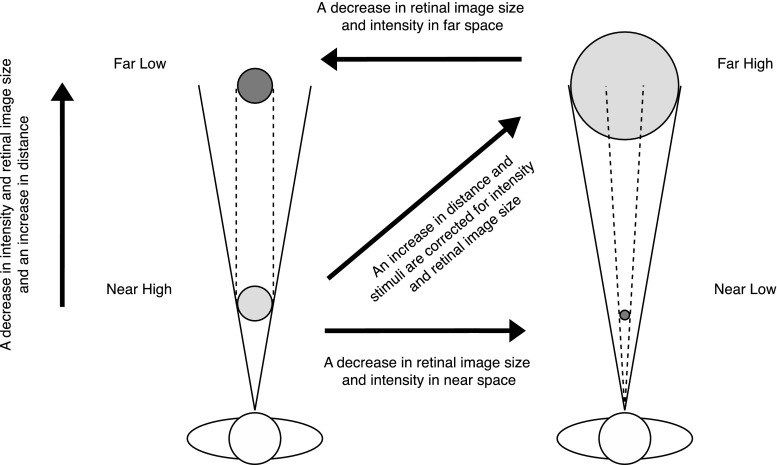
Schematic overview of the four conditions that were used in the experiment. Note that the retinal image size and the brightness of the visual stimulus, as well as the sound pressure level of the auditory stimulus at the observer’s location, were the same in the *Near High* intensity and *Far High* intensity conditions and in the *Near Low* intensity and the *Far Low* intensity conditions

To project visual stimuli in near and far space, we used an Acer X1261P projector (60 Hz) that was placed behind the observer projecting down in a small angle. The projection screen in near space was located at a distance of ~80 cm from the observer and at ~208 cm in far space. These distances were chosen as they were considered to be within the reachable (near/peripersonal space) and unreachable space (far/extrapersonal space) that were discussed in the “Introduction” section. In previous studies, it has been shown that perceptual processing can differ depending on the region of space (peripersonal vs. extrapersonal) in which the stimuli are presented (e.g., Aimola et al. 2012; Halligan and Marshall 1991; Occelli et al. 2011; Previc 1998; Van der Stoep et al. 2013; Van der Stoep et al. 2014). When stimuli were presented in far space, the projection screen in near space was placed out of sight. Six speakers (Harman/Kardon HK206, Frequency response: 90–20,000 Hz) were used to present auditory stimuli at three locations (left, center, right) in both near and far space (resulting in six locations). An overview of all conditions is shown in Fig. 1.

**Fig. 2:**
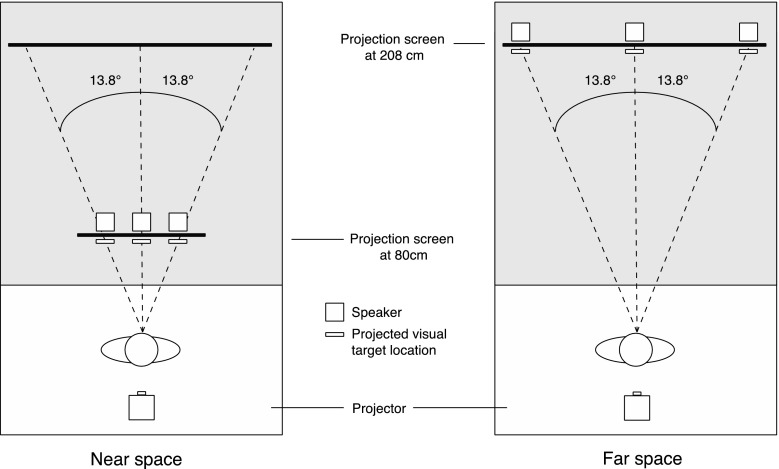
Schematic bird’s-eye view of speaker and projected target locations in near space (*left panel*) and far space (*right panel*) setup

The auditory stimuli consisted of 100-ms white noise bursts (with a 15-ms rise and fall of the signal). In the *Near High* and the *Far High* condition, auditory stimuli were presented at ~70 dB(A) SPL as measured from the location of the participant. In the *Near Low* and *Far Low* condition, auditory stimuli were presented at ~60 dB(A) SPL as measured from the location of the participant. This difference in intensity was chosen as the increase in distance between the speaker and the observer resulted in a decrease of approximately 10 dB(A) SPL. The reverberant room was 2.60 m wide, 5.10 m long, and 2.80 m high. The difference in the direct sound and the sound reflections between near and far space provided a cue for the distance of the sound. When the auditory stimulus was corrected for intensity, the differing reflections between near and far space provided information about the distance of the stimulus [see supplementary Figure 1 for the impulse response of sound presented at the central location (see Fig. 2) in near (80 cm; S1 top) and far space (208 cm; S1 bottom)]. The visual stimuli consisted of a gray-filled circle (*Near High* and *Far High*: 3° in diameter, ~6.6 cd/m^2^, *Near Low* and *Far Low*: 1.16° in diameter, ~1.98 cd/m^2^; intensities were measured from the location of the participant using a PhotoResearch SpectraScan PR 650 spectrometer) that was presented on a dark gray background (near and far space: ~1.3 cd/m^2^). The fixation cross was a gray plus sign (near and far space: 0.7° × 0.7°, ~6.6 cd/m^2^). Visual target locations were 13.8° to the left and right of fixation, and in the center of the screen at the location of the fixation cross. The speakers were placed directly behind the locations of the projected visual stimuli in both near and far space to ensure perfect spatial alignment. Audiovisual stimuli were always presented spatially and temporally aligned. A schematic bird’s-eye view of the experimental setup is shown in Fig. 2.

The experiment consisted of two blocks: 360 trials in near space and 360 trials in far space. The distance from which stimuli were presented was blocked and counterbalanced across participants. Stimuli originating from the same distance were all presented in a single block. There were two intensity conditions for each distance resulting in a *Near High*, a *Near Low*, a *Far High*, and a *Far Low* intensity condition. Within each distance block, two breaks were introduced: one after 120 trials and another one after 240 trials. Participants were able to continue the experiment by pressing the space bar. Both near and far blocks contained 180 high-intensity and 180 low-intensity trials. Each of those 180 trials consisted of 60 auditory, 60 visual, and 60 audiovisual targets, and each of those 60 trials contained 20 trials in which the target was presented to the left of the fixation cross, 20 trials to the right of the fixation cross, and 20 trials presented at the location of the fixation cross.

### Procedure

The experiment was conducted in a room where the light from the projector was the only source of illumination. Participants were asked to place their chin in a chinrest. Before the start of the experiment, participants had to report the location of three auditory stimuli (white noise) that were presented at three different locations in near space (left, center, and right) as a quick test to confirm their ability to localize these sounds. All participants were able to correctly report the locations of the auditory stimuli.

Each trial started with the presentation of the fixation cross. After a random duration between 750 and 1250 ms, the fixation cross disappeared, and a blank screen was presented. After another random duration between 200 and 250 ms, either an auditory, visual, or audiovisual target was presented for 100 ms after which the target disappeared. The target modality, location, and intensity were randomized across trials. Participants were instructed to press a button on a response box as soon as they detected either a visual, auditory, or audiovisual stimulus to the left or the right side of the fixation cross (Go trials), but to withhold their response when a stimulus was presented in the center (No-go trials). Given that participants only had to respond to lateral targets, but not to central targets in the experiment, their ability to correctly localize the auditory stimuli was reflected by the amount of errors made in responding to auditory targets (i.e., whether they were able to differentiate between auditory stimuli presented to the lateral locations and the central location). The response window was 2,000 ms from target onset.

### Data analysis

Go trials with response times between 100 and 1000 ms and No-go trials without a response were considered correct. Only response times on Go trials between 100 and 1000 ms were analyzed, because responses faster than 100 ms were considered to be the result of anticipation and responses slower than 1000 ms the result of not paying attention to the task. This led to the removal of 2.60 % of the data in the near space conditions and 2.53 % of the data in far space. In the near space condition, 0.77 % of the Go trials (High = 0.37 %, Low = 1.17 %) and 6.25 % of the No-go trials (High = 7.25 %, Low = 5.25 %) were removed, and in far space, 0.65 % of the Go trials (High = 0.50 %, Low = 0.79 %) and 6.29 % of the No-go trials (High = 6.92 %, Low = 5.67 %) were removed. The median response times of each participant in each condition were used in the RT analysis as RT distributions are generally skewed and the median is less affected by the presence of outliers.

A 2 × 3 × 2 repeated-measures ANOVA with the factors Target Space (Near, Far), Target Modality (A, V, AV), and Intensity (High, Low) was used to analyze RTs. The Greenhouse–Geisser correction was used whenever the assumption of sphericity was violated. To test for differences in RT between conditions, we used two-tailed paired samples *t* tests (*p* values were corrected using the Bonferroni method where indicated: corrected *p* value = *p* × number of tests).

To investigate the increase in the speed of detection in the audiovisual condition compared to the fastest unimodal condition, we calculated the amount of absolute multisensory response enhancement (aMRE) and the amount of relative multisensory response enhancement (rMRE) for each condition (*Near High*, *Near Low*, *Far High*, and *Far Low*):

$${\text{absolute}}\, {\text{MRE}} = \hbox{min} \left( {{\text{RT}}_{\text{A}} , {\text{RT}}_{\text{V}} } \right) - {\text{RT}}_{\text{AV}}$$

$${\text{relative}}\, {\text{MRE}} = \frac{{\hbox{min} \left( {{\text{RT}}_{\text{A}} , {\text{RT}}_{\text{V}} } \right) - {\text{RT}}_{\text{AV}} }}{{\hbox{min} \left( {{\text{RT}}_{\text{A}} , {\text{RT}}_{\text{V}} } \right)}} \times 100\,\%$$

Note that the median RT of each condition was used to calculate the amount of aMRE and rMRE for each participant. The amount of rMRE was also calculated because of possible unimodal baseline differences in the different intensity conditions (low and high) and the different space conditions (near and far). Two-tailed planned pairwise comparisons were used to test for the differences between the *Near High*, *Far High*, *Near Low*, and *Far Low* conditions.

To test whether the observed response enhancement for audiovisual targets could be explained by statistical facilitation, the cumulative distributive function (CDF) of RTs to auditory, visual, and audiovisual targets was calculated for each condition (*Near High*, *Near Low*, *Far High*, and *Far Low*). Using these CDFs, the upper bound of statistical facilitation predicted by a race model was calculated using the race model inequality (Raab 1962; Miller 1982, 1986; Ulrich et al. 2007):

$$P\left( {{\text{RT}}_{\text{AV}} < t} \right) < = P\left( {{\text{RT}}_{\text{A}} < t} \right) + P\left( {{\text{RT}}_{\text{V}} < t} \right)$$

The race model inequality represents the probability (*P*) of a given RT in the audiovisual condition that is less than or equal to a given time *t* in milliseconds based on the combined probabilities for a given RT in the unimodal conditions where *t* ranges from 100 to 1000 ms (assuming a maximum negative correlation of −1 between detection times of unimodal stimuli).

The performance in the audiovisual condition can be compared to the upper bound predicted by the race model inequality in two ways. First, RTs for a range of quantiles (i.e., 10, 20, up to 90 %) of the CDF can be compared between the audiovisual CDF and the race model CDF. Second, probabilities between the audiovisual CDF and the race model CDF can be compared for a range of RTs. Shorter RTs or larger probabilities in the audiovisual condition compared to the race model indicate MSI. Comparing RTs at a range of quantiles reveals how much shorter RTs in the audiovisual condition are in an absolute sense (i.e., difference in ms) at comparable points of the audiovisual and race model CDF. In addition, the difference in probability indicates in which RT range differences between the AV CDF and the race model CDF occur. Given that both methods provide different information and have (in isolation) been used in previous studies (e.g., Girard et al. 2013; Stevenson et al. 2012; Van der Stoep et al. 2015), both methods were used to analyze the data in the current study.

Differences in RT between the audiovisual CDF and the race model CDF for each quantile (i.e., race model inequality violation) were analyzed using one-tailed pairwise comparisons for each quantile and each condition (*p* values were corrected for nine tests in each condition using the Bonferroni method). Differences in probability between the audiovisual CDF and the race model CDF for each RT point were analyzed using paired samples *t* tests.

Four additional measures were extracted from the race model inequality violation curves in each condition of each participant: (1) the mean race model inequality violation value across nine quantiles, (2) an estimate of the area under the race model inequality violation curve that was based on differences in RT for each quantile, (3) the maximum race model inequality violation in terms of probability, and (4) the corresponding time point of this maximum race model inequality violation. These values were extracted for each condition and each participant and compared between conditions using planned pairwise comparisons.

## Results

### Accuracy

Overall accuracy was very high (overall *M* = .965, SE = .005, Go trials: *M* = .993, SE = .002, No-go trials: *M* = .937, SE = .010) indicating that participants were well able to localize and detect targets, and withhold their response when targets appeared at the central location.

### Response times

**Fig. 3:**
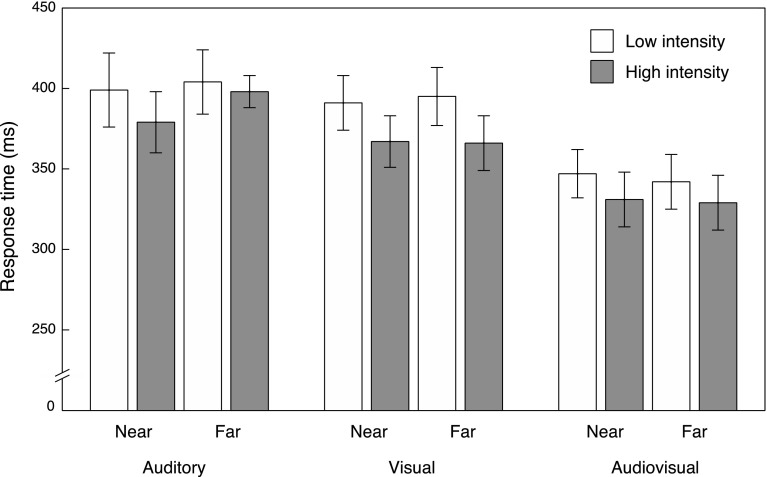
Average of median response times to auditory, visual, and audiovisual targets in near and far space for the low (*white*) and high (*grey*) intensity condition. *Error bars* represent SE of the mean

A significant main effect of Target Modality [*F*(2, 38) = 47.048, *p* < .001, *η*_partial_^2^ = .712] was observed. The RTs to audiovisual targets (*M* = 337 ms, SE = 16) were significantly shorter compared to visual (*M* = 380 ms, SE = 16, *t*(19) = 8.655, *p* < .001, *d* = .6) and auditory targets (M = 395 ms, SE = 19, *t*(19) = 9.337, *p* < .001, *d* = .591). The difference between RTs to visual and auditory targets was not significant [*t*(19) = .136, *p* = .138]. The average median RTs for each condition are shown in Fig. 3.

There was also a main effect of Intensity [*F*(1, 19) = 61.304, *p* < .001, *η*_partial_^2^ = .763]. RTs to high-intensity stimuli (*M* = 362 ms, SE = 17) were significantly shorter compared to RTs to low-intensity stimuli (*M* = 380 ms, SE = 17). No main effect of Target Space was apparent [*F*(1, 19) = .098, *p* = .758, *η*_partial_^2^ = .005].

The interaction between Target Modality and Target Space was also significant [*F*(2, 38) = 4.400, *p* = .019, *η*_partial_^2^ = .188]. This effect appeared to be driven by a larger difference in RTs between auditory and visual targets in far as compared to near space (mean difference near space = 10 ms, SE = 8, mean difference far space = 21 ms, SE = 7). The difference in RTs to auditory and visual targets was significant in far space [*t*(19) = −2.792, *p* = .036, *d* = −.232], but not in near space [*t*(19) = 1.282, *p* = .645]. One could argue that the difference between auditory and visual RTs is simply the result of stimulus characteristics. After all, at a distance of 208 cm, the auditory stimulus reaches the observer approximately 6.06 ms later than the visual stimulus. Indeed, if this delay in arrival time is subtracted from the RTs to auditory stimuli in far space, the RT difference between auditory and visual targets in far space ceases to be significant [*t*(19) = −1.924, *p* = 0.192].

The interaction between Target Modality and Intensity was also significant [*F*(1.491, 28.323) = 5.280, *p* = .018, *ε* = .745, *η*_partial_^2^ = .217]. The difference in RTs between auditory and visual targets was significant for high-intensity stimuli (*M* Auditory High = 388 ms, SE = 19 vs. *M* Visual High = 366 ms, *SE* = 16, *t*(19) = 3.537, *p* = .004, *d* = .250], but not for low-intensity stimuli (*M* Auditory Low = 402 ms, SE = 20 vs. *M* Visual Low = 393 ms, SE = 16, *t*(19) = 0.972, *p* = .686). The comparisons between RTs to auditory and audiovisual, and visual and audiovisual targets were significant for both the high and low intensity conditions (all *t*’s > 7, all *p*’s < .001, also see the “Multisensory response enhancement” section below). Furthermore, responses to high-intensity targets were faster as compared to low-intensity targets, as could be expected based on the intensity manipulation. The difference in RT between the high and low intensity conditions was significant for auditory (*M* difference = 13 ms, SE = 5), visual (*M* difference = 27 ms, SE = 3), and audiovisual targets (*M* difference = 14 ms, SE = 2); all *t*’s < −2.7, all *p*’s < .05). The difference in RT between the high and low intensity conditions was slightly larger for visual targets as compared to auditory and audiovisual targets.

The interaction between Target Space and Intensity [*F*(1, 19) = .705, *p* = .412, *η*_partial_^2^ = .036], and the three-way interaction between Target Modality, Target Space, and Intensity were not significant [*F*(2, 38) = 1.053, *p* = .359, *η*_partial_^2^ = .052].

In sum, we observed a general increase in the speed of responses in the audiovisual condition compared to the unimodal conditions, and high-intensity stimuli evoked faster responses compared to low-intensity stimuli. Overall, responses to auditory stimuli were slightly slower when presented in far space compared to near space, but this effect could be explained in terms of differences in arrival times. To investigate whether our correction for stimulus intensity and size across distance for both auditory and visual stimuli resulted in similar response times between the near and far condition, we compared RTs in the *Near High* and *Far High*, and the *Near Low* and *Far Low* conditions for auditory and visual targets. If the distance from which stimuli were presented had no particular influence on RTs per se, then no difference between these conditions should be observed. Indeed, we did not find any significant differences between the Near and Far conditions for neither low nor high stimulus intensities for auditory and visual targets (all *t*’s < 1.7, all *p*’s > .1).

### Multisensory response enhancement

**Fig. 4:**
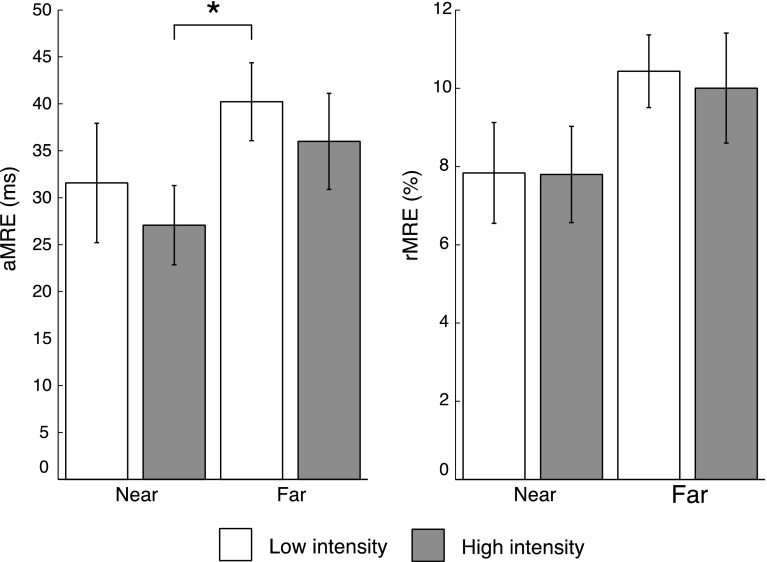
*Left panel* the average amount of aMRE in each condition. *Right panel* the average amount of rMRE in each condition. *Error bars* indicate SE of the mean. Significant differences are indicated with an *asterisk*

One-sample *t* tests confirmed that there was a significant amount of absolute and relative MRE in each condition (all *t*’s > 4.9, *p*’s < .001, in the *Near Low*, *Near High*, *Far Low*, *Far High* Intensity condition), indicating that responses to multisensory targets were significantly faster when compared to the fastest response to either of the unimodal targets. The average aMRE and rMRE in each condition are shown in the left and right panel of Fig. 4, respectively.

When the audiovisual stimulus was moved from near to far space, while keeping the stimulus properties the same (i.e., decreased intensity and retinal image size in the far space condition as measured from the location of the participant: *Near High* vs. *Far Low*), the average amount of aMRE was significantly larger in far space (*M* *Far Low* = 40 ms, SE = 4) as compared to near space (*M* *Near High* = 27 ms, SE = 4, *t*(19) = −2.209, *p* = .040, *d* = −.702). To investigate whether this increase in aMRE was the result of a decrease in retinal image size and stimulus intensity alone or whether the distance or the region of space from which the stimuli were presented also contributed to the effect, the amount of aMRE in the High intensity and the Low intensity conditions in near space was also compared. Interestingly, there was no difference in the amount of aMRE between the *Near High* and the *Near Low* intensity condition (*M* *Near High* = 27 ms, SE = 4 vs. *M* *Near Low* = 32 ms, SE = 6, *t*(19) = .742, *p* = .467). These results indicate that a decrease in stimulus intensity and retinal image size alone did not increase the amount of aMRE.

One could also argue that the difference in the distance from which the stimuli were presented is the only factor that contributes to the increase in aMRE in far space. Therefore, the amount of aMRE was also compared between the *Near High* and *Far High* condition in which the far space stimuli were corrected for intensity and retinal image size (*M* *Near High* = 27 ms, SE = 4 vs. *M* *Far High* = 36 ms, SE = 5). A significant difference between the Far Low and the Near Low condition would indicate that a change in distance alone could explain an increase in aMRE, but this was not the case [*t*(19) = −1.518, *p* = .145]. To check whether a decrease in intensity resulted in a generally larger amount of aMRE in far space, the *Far High* and *Far Low* conditions were compared, but the difference was also not significant [*t*(19) = .757, *p* = .458].

Thus, the decrease in stimulus intensity and retinal image size alone could not explain the observed pattern of aMRE, a result that does not seem to be in line with the principle of inverse effectiveness, but the use of only two intensities make it difficult to draw firm conclusions about this. Interestingly, the distance from which the stimuli were presented could also not explain these results, which suggests that aMRE is especially increased when an increase in the distance from which stimuli are presented and a decrease in retinal image size and stimulus intensity co-occured.

The rMRE was analyzed in the same way as the aMRE was. A similar, but less pronounced, effect as with the aMRE measure was observed for the rMRE measure (Fig. 4 right panel). The difference between the *Near High* (*M* = 7.8 %, SE = 1.2) and *Far Low* condition (*M* = 10.4 %, SE = 0.9) was not significant [but in the right direction, *t*(19) = −1.852, *p* = .080, *d* = −.402]. An effect size of −.548 could be considered medium to large, which may indicate a lack of power. As in the aMRE measure, the other comparisons were not significant: *Near High* versus *Near Low* [*M* = 7.8 %, SE = 1.3, *t*(19) = −.030, *p* = .976], *Near High* versus *Far High* [*M* = 10.0 %, SE = 1.4, *t*(19) = −1.514, *p* = .147], and *Far High* versus *Far Low* [*t*(19) = −.289, *p* = .776]. The results of the rMRE analysis showed a similar pattern as the results of the aMRE analysis, but the critical difference, namely the difference between the *Near High* and the *Far Low* condition, failed to reach significance (*p* = .080). Both the absolute and relative MRE measures were reported here because it has been shown that a pattern of inverse effectiveness can depend on whether an absolute or a relative measure of multisensory response enhancement is analyzed (see for example Holmes 2007, for a discussion). Although it is unclear as to why the rMRE measure only showed a trend regarding the distance-based inverse effectiveness effect in the current study (*p* = .080), both the aMRE and the rMRE measure showed a similar pattern.

### Race model inequality violation

**Fig. 5:**
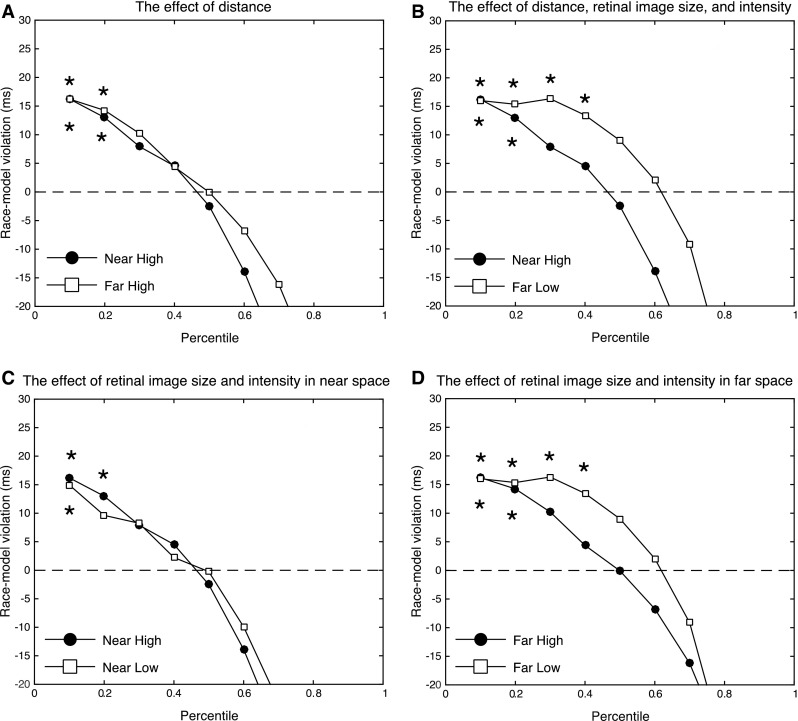
Difference curves show the amount of race model inequality violation in each condition in terms of response time differences across the range of percentiles. **a** The effect of distance on race model inequality violation, **b** the effect of distance combined with a decrease in intensity and retinal image size, **c** the effect of a decrease in intensity and retinal image size in near space, and **d** the effect of a decrease in intensity and retinal image size in far space. *Asterisks* indicate *p* values that were smaller than .05 after correction for multiple tests using the Bonferroni method

To investigate whether multisensory response enhancement could be explained in terms of statistical facilitation alone (i.e., the race model) or better by MSI, the audiovisual CDF was compared to the race model CDF in each condition using paired samples *t* tests at nine points of the CDF. We compared both differences in RT across a range of quantiles (see Fig. 5) and differences in probability across a range of RTs based on the full CDF function (see Fig. 7).

The comparison between differences in RT at corresponding quantiles revealed significant violations at the 10th percentile in the *Near Low* condition. In the *Near High* condition, we observed significant violations at the 10th and the 20th percentile. Interestingly, in the Far Space Low Intensity condition (i.e., the same stimulus as in the *Near High* condition, but presented at a larger distance), we observed race model inequality violations over a broader range of percentiles, from the 10th to the 40th percentile. In the Far Space High Intensity condition, significant violations of the race model inequality were found at the 10th and the 20th percentile (for all four conditions: *t*’s > 2.9, *p*’s < .05, one-tailed, corrected using the Bonferroni method).

**Fig. 6:**
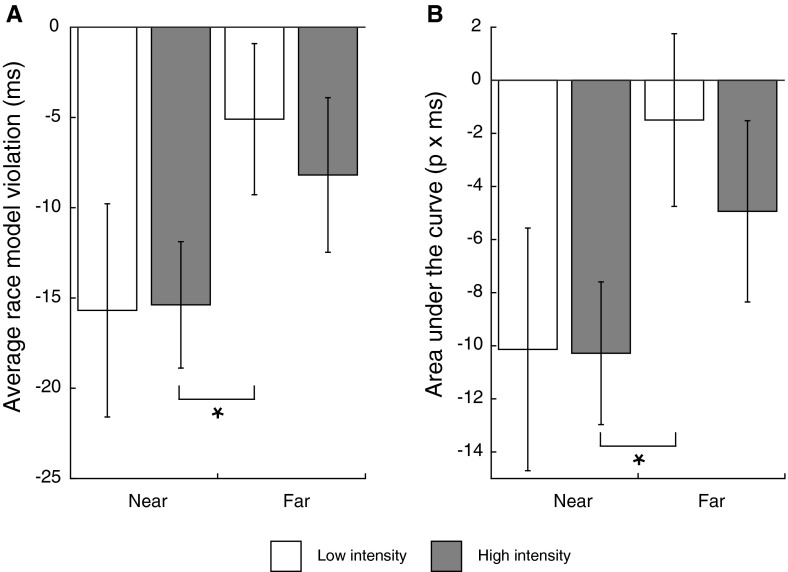
**a** Average amount of race model inequality violation in each condition. **b** The average area under the curve in each condition (*right panel*). More positive values reflect a larger amount of race model inequality violation. Significant differences are indicated with an *asterisk* (*p* < .05)

The average amount of race model inequality violation and the average net area under the curve for each condition are shown in the left and right panel of Fig. 6, respectively. The race model CDF is very steep compared to a normal CDF as its probability sums up to 2 (and is cut of at 1). Therefore, race model inequality violations are almost always negative at the higher percentiles of the RT distribution, which leads the average amount of violation to be negative on average in all four conditions. Nevertheless, differences between conditions still reflect differences in overall race model inequality violation. As with the amount of aMRE, the average amount of race model inequality violation was larger when the stimulus was moved further away from the participants while the stimulus properties remained constant [*M* *Near High* = −15.382, SE = 3.501 vs. *M* *Far Low* = −5.097, SE = 4.187, *t*(19) = −2.302, *p* = .033, *d* = −.594]. Interestingly, the same decrease in retinal image size and intensity did not result in an increase in the amount of race model inequality violation when stimuli were presented at the same distance [*M* *Near High* = −15.382, SE = 3.501 vs. *M* *Near Low* = −15.688, SE = 5.906, *t*(19) = −.069, *p* = .946, *d* = −.013]. Presenting the audiovisual stimulus at a larger distance, but correcting for retinal image size and intensity, did not result in a larger amount of race model inequality violation [*M* *Near High* = −15.382, SE = 3.501 vs. *M* *Far High* = −8.188, SE = 4.282, *t*(19) = −1.638, *p* = .118, *d* = −.409]. The difference between the *Far High* and the *Far Low* intensity condition was not significant [*M* *Far Low* = −5.097, SE = 4.187 vs. *M* *Far High* = −8.188, SE = 4.282, *t*(19) = .586, *p* = .565, *d* = .163], indicating that a decrease in intensity and retinal image size did not result in larger race model inequality violations in far space.

The results of the signed area under the curve measure were the same as the average amount of race model inequality violation. The average net area under the curve was more positive in the *Far Low* condition compared to the *Near High* condition (*M* *Near High* = −10.282, SE = 2.687 vs. *M* *Far Low* = −1.499, SE = 3.253, *t*(19) = −2.487, *p* = .022, *d* = −.656), indicating that audiovisual integration was enhanced when the audiovisual stimulus was presented in far space compared to a stimulus that was physically the same, but was presented in near space. None of the other comparisons were significant (0 > *t*’s > −1.8, all *p*’s > .1).

### The response time range of race model inequality violations

**Fig. 7:**
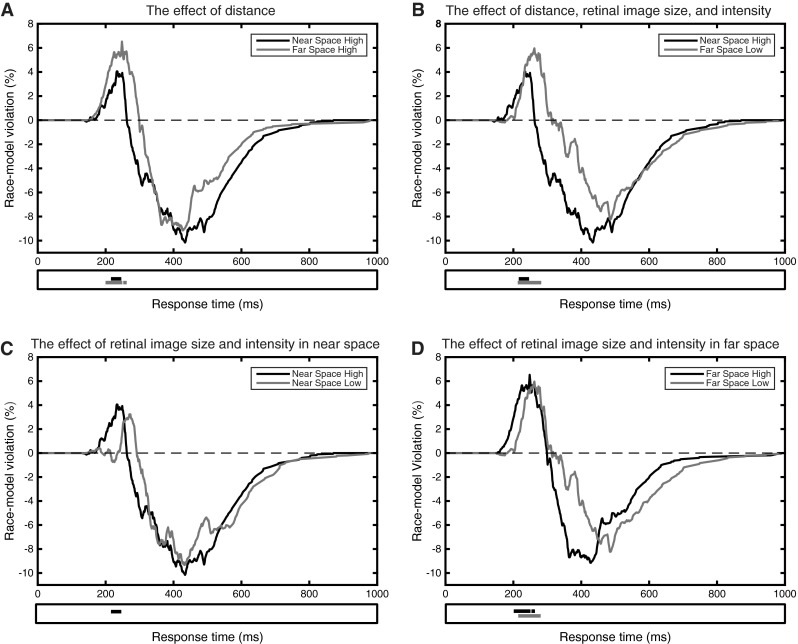
Difference curves that show the amount of race model inequality violation in each condition in terms of differences in probability across the full range of time points. **a** The effect of an increase in distance on race model inequality violation, **b** the effect of an increase in distance combined with a decrease in intensity and retinal image size, **c** the effect of a decrease in intensity and retinal image size in near space, and **d** the effect of a decrease in intensity and retinal image size in far space. The *bars* below each graph along the *x*-axis indicate significant positive violations of the race model inequality (*p* < .05, uncorrected)

Comparing the probabilities between the audiovisual CDF and the race model at a range of RTs (100–1000 ms) revealed the RT range in which the race model was violated (see Fig. 7). Only positive deviations from the race model were of interest (see the bar below each graph in Fig. 7), as the race model inequality provides an upper bound of facilitation, not a lower bound. We observed significant violations in the *Near High* condition between 219 and 243 ms (range 25 ms, see panels a, b, or c of Fig. 7). In the *Near Low* condition, there were no statistically significant race model inequality violations in terms of differences in probability (see panel c of Fig. 7).^1^ The range of RTs in which violations occurred in the *Far Low* condition was from 216 to 282 ms (range 67 ms, see panel b or d of Fig. 7), and from 203 to 249 ms (range 47 ms) and 255–262 ms in the *Far High* condition (range 8 ms, see panel a or d of Fig. 7).

The RT range analyses indicate that an increase in distance mainly resulted in an increase in the width of the range in which race model inequality violations occurred, from a range of 25 ms in the *Near High* condition to an average range of 47 ms in the *Far High* condition (compare the gray and black bars in panel a of Fig. 7). A decrease in intensity and retinal image size mainly resulted in a shift of the RT range of significant race model inequality violations to longer RTs (compare the gray and black curves in panel c and d of Fig. 7). Race model inequality violations were significant starting with RTs larger than 219 ms in the *Near High* condition. Although no significant violations of the race model inequality in terms of probability differences were observed in the *Near Low* condition, a clear shift to later RTs can be seen in Fig. 7C. In the *Far High* condition, race model inequality violations were significant starting from RTs larger than 203 and from 216 ms in the *Far Low* condition (a shift of 13 ms).

The observed stronger MSI when the same stimulus was presented in far space (~200 cm) could thus be explained by the combined effects of an increase in distance on the one hand, and a decreased retinal image size and intensity on the other. This was apparent in larger race model inequality violations (see Figs. 6, 7), and also a larger range in which race model inequality violations occurred in terms of both quantiles (Fig. 5) and a broader RT range (Fig. 7).

### Maximum race model inequality violation and shifts in the time point of the maximum violation

To further explore differences in race model inequality violation, the maximum probability difference and the corresponding time point at which these maximum race model inequality violations occurred were compared between the conditions (see Table 1). Note that the average maximum probability difference reported here does not correspond with the observed maximum probability differences in Fig. 7. The difference curves that are depicted in Fig. 7 are averaged across subjects. As each subject showed race model inequality violations at slightly different time points, the average difference curves do not show the same maximum probability difference [that is why we also analyzed differences in RT for comparable points (quantiles) on the CDFs of each participant and each condition, see Fig. 5].

**Table 1:** Average maximum probability difference between the AV CDF and the race model, and the average corresponding time point of the maximum race model inequality violation in each condition

Condition	Max. violation (%)	Time point of max. (ms)
Near space high intensity	14.03 (1.83)	285 (14)
Near space low intensity	14.10 (2.07)	295 (22)
Far space high intensity	16.74 (2.56)	286 (13)
Far space low intensity	18.45 (2.17)	319 (16)

The SE are presented between brackets

There were no significant differences in the average maximum race model inequality violation between the conditions. There was, however, a significant difference in the time point of the maximum race model inequality violation between the *Near High* and the *Far Low* condition [*M* *T*_Near High_ = 285 ms, SE = 14, *T*_Far Low_ = 319 ms, SE = 16, *t*(19) = −2.910, *p* = .009, *d* = −.506]. The other differences in time points of the maximum race model inequality violation were not significant (0 > *t*’s > −1, all *p*’s > .5).

A decrease in retinal image size and intensity seemed to cause a shift of the time point of the maximum amount of race model inequality violation to longer RTs. The increase in the distance between stimuli and the observer seemed to slightly increase the average maximum amount of race model inequality violation, but these differences were not statistically significant. Only when stimuli were presented from far space and were of smaller retinal image size and intensity, did the shift in time point change significantly.

## Discussion

Multisensory integration is generally assumed to be enhanced for weakly effective stimuli as compared to strongly effective stimuli (i.e., the principle of inverse effectiveness) (e.g., Holmes 2007, 2009; Leone and McCourt 2013; Meredith and Stein 1983). Yet, conflicting findings have been reported with respect to the principle of inverse effectiveness. What these studies have in common is that stimuli were always presented in a single depth plane. Given that increases in the distance between auditory and visual stimuli from the observer are generally related to decreases in retinal image size and intensity, audiovisual integration may be especially enhanced when decreases in retinal image size and intensity co-occur with increases in distance.

Our findings indicate that a decrease in retinal image size and stimulus intensity resulted in stronger audiovisual integration, but only when the stimulus was also presented at a larger distance from the observer (i.e., in far space). One could argue that moving a stimulus further away while keeping the stimulus physically the same causes an increase in audiovisual integration because of the principle of inverse effectiveness. This was, however, not the case, as the same decrease in intensity and retinal image size without an increase in distance did not result in enhanced audiovisual integration. Furthermore, the observed enhanced audiovisual integration in far space could not be explained solely based on the region of space in which the stimuli were presented, as an increase in the distance, while correcting the stimuli for intensity and retinal image size, did not significantly increase audiovisual integration.

A thorough analysis revealed how changes in retinal image size and intensity and changes in distance contributed to an increase in audiovisual integration in far space as compared to near space. A decrease in retinal image size and intensity without a change in the distance from which the stimuli were presented mainly resulted in a shift of the RT range to longer RTs in which race model inequality violations occurred. An increase in the distance while keeping the stimulus intensity and retinal image size constant caused an increase in the width of the RT range in which race model inequality violations occurred. Interestingly, however, the combination of these factors resulted in an effect that was different from the sum of these effects; an increase in the distance, and a decrease in stimulus intensity and retinal image size resulted in both an increase in the amount of race model inequality violation and an increase in the RT range in which violations occurred. It did not, however, result in a shift of the RT range in which violations occurred to longer RTs in far as compared to near space.

The current results thus indicate that stimulus efficacy and stimulus distance interact to increase audiovisual integration in far space. Changes in retinal image size and stimulus intensity alone did not increase audiovisual integration when the distance remained the same. However, given that only two retinal image sizes and stimulus intensities were used, which were also above threshold, it is difficult to take the current results as evidence against a general principle of inverse effectiveness. Although an increase in distance or a decrease in stimulus efficacy might independently affect audiovisual integration when large differences in distance or stimulus efficacy are used, the current findings indicate that their combined effects specifically enhance audiovisual integration at the distances used here (near: 80 cm, far: 208 cm). A comparison between supra-threshold stimuli and near-threshold stimuli in relation with distance-dependent changes in stimulus efficacy would require the presentation of stimuli at very large distance (this will also affect the differences in arrival times more strongly).

Multisensory interactions involving stimulation of the skin are often more pronounced when the source of multisensory stimulation is presented in peripersonal space as compared to extrapersonal space (see Van der Stoep et al. 2014 for a review). As auditory and visual perception does not depend on the distance between a stimulus and the body, no spatial asymmetry in depth is expected in terms of the strength of audiovisual interactions. Indeed, we did not observe any difference in audiovisual integration between information presented in near and far space when the audiovisual stimulus was corrected for intensity and retinal image size.

As for the cause of the current effects, there might be a role for multisensory experience. Several neurophysiological studies have shown the importance of prior experience with the environment in shaping the way the brain responds to multisensory stimuli (e.g., Wallace and Stein 1997, 2001, 2007). This is further underlined by results from behavioral studies in humans that indicate, for example, that the perceived temporal alignment of multisensory stimuli can be recalibrated based on prior multisensory experience (e.g., Vroomen et al. 2004; Machulla et al. 2012). A factor that changes the way we perceive multisensory information on a daily basis is the distance between audiovisual stimuli and the body. Typically, increasing the distance between stimuli and the observer results in a decrease in retinal image size and stimulus intensity when the stimulus properties of the source remain constant. Given that this relation is encountered in our everyday multisensory experiences, it might shape the way the brain responds to audiovisual stimuli. It has also been suggested that audition and vision are especially important for spatial orienting in far space (Previc 1998). As a result of this distance-related multisensory experience, one might argue that relatively weak audiovisual stimuli in far space may be more helpful in spatial orienting as compared to the same stimuli when presented in near space. The observation of enhanced audiovisual integration in far space as compared to near space in a localization detection task is in line with this idea.

Another (underlying) factor that may contribute to the observed effects may be found in differences in spatial uncertainty of audiovisual sources between near and far space. When comparing the retinal image size of, for example, a car in near space and far space, it is evident that the car covers a larger visual angle in near space as compared to far space. At a large distance, several cars could be observed in the same visual angle as that of the car in near space. Speculatively, audiovisual integration may be generally more helpful in spatial orienting in far space, when compared to near space, as the spatial uncertainty of the location of both visual and auditory information is higher (in this case the car).

The idea that spatial uncertainty enhances audiovisual integration is in line with the results from previous studies, in which it was shown that MSI is enhanced when the location of a multisensory target is unattended as compared to when it was attended (exogenous spatial attention: Van der Stoep et al. 2015; endogenous spatial attention: Zou et al. 2012). Although seemingly unrelated to the explanation of our findings in terms of the uncertainty of the spatial location of stimuli, these studies demonstrated that MSI might not help as much during spatial localization when attention is already close to or at the target location as compared to when spatial attention is at a location that is far from the target location. In other words, MSI is stronger when spatial uncertainty is larger. In the current study, the broader window during which MSI occurred in far space as compared to near space may reflect a tendency for the brain to accumulate as much evidence as possible for the spatial location of the stimuli in far space because of a larger spatial uncertainty.

In the current study, the distance from which the target stimulus was presented was blocked. Therefore, participants could focus their attention endogenously at a certain depth, reducing spatial uncertainty in the depth space. It might be interesting in future research to investigate how the uncertainty of audiovisual information in depth affects audiovisual integration in near and far space. This may also depend on where attention is focused in terms of depth as some studies have observed faster responses to stimuli occurring between the body and the focus of attention, compared to stimuli presented beyond the focus of attention (e.g., de Gonzaga Gawryszewski et al. 1987). The current findings indicate that when endogenous attention is focused at the depth from which information is presented, audiovisual integration is enhanced in far space relative to near space when the stimulus is not corrected for retinal image size and intensity (i.e., a smaller retinal image size and a lower visual and auditory intensity in far space as compared to near space).

To conclude, we observed stronger audiovisual integration in far space when stimuli were not corrected for retinal image size and intensity as compared to the same audiovisual stimuli in near space. The increase in integration could not be explained in terms of inverse effectiveness alone, as the same decrease in retinal image size and intensity did not result in an increase in audiovisual integration in near space. The presentation of audiovisual stimuli in far space did seem to slightly increase the amount of audiovisual integration, but only when an increase in distance was combined with a decrease in retinal image size and intensity, was the enhanced integration in far space significantly different from that in near space. These results underline the importance of taking the distance from which information is presented into account when investigating multisensory interactions.

## Electronic supplementary material

Below is the link to the electronic supplementary material.

Supplementary material 1 (EPS 904 kb)

## Footnotes

1 When a different form of the race model inequality was used which does not assume a maximal negative correlation of −1 between signals [*P*(RT_AV_ <= *P*(RT_A_) + *P*(RT_V_) − *P*(RT_A_) × *P*(RT_V_)], violations were observed from 267 to 285 ms (range 18 ms).
